# Editorial: Bacteriophages to treat infections with multidrug resistant pathogens

**DOI:** 10.3389/fmed.2023.1348463

**Published:** 2024-01-05

**Authors:** Dinesh Subedi, Tang Fang, Ram Prasad Bhusal, Mark Willcox

**Affiliations:** ^1^School of Biological Sciences, Monash University, Clayton, VIC, Australia; ^2^College of Veterinary Medicine, Nanjing Agricultural University, Nanjing, China; ^3^Department of Biochemistry and Molecular Biology, Monash Biomedicine Discovery Institute, Monash University, Clayton, VIC, Australia; ^4^School of Optometry and Vision Science, University of New South Wales, Sydney, NSW, Australia

**Keywords:** bacteriophage (phage), phage therapy, antimicrobial resistance (AMR), bacteria, infections

This Research Topic was designed by us in response to the growing threat of antibiotic resistance of bacterial infections worldwide. The use of bacteriophages (phages) as alternatives or adjunct agents to treat infections is gaining wider use. Indeed, phage therapy has been successfully used to treat patients across a broad range of pathologies, with substantial clinical improvement and bacterial eradication (1). However, we noted that only a few clinical requests for phage therapy have been fulfilled successfully due to a lack of understanding of the biology of phage-host interactions and resistance development (2), the human immune response to phage particles (3), the right phage dosage and pharmacokinetics (4). Bacterial defenses such as, restriction-modification and CRISPR-Cas system, also contribute to abortive infections by modifying phage DNA (5).

At the conclusion of the Research Topic, 12 articles have been published that address critical and interesting issues. There were six original or brief research reports, five reviews or mini reviews and one case report. There were reviews of phage therapy; studies on phages active against *Shigella* spp., *Salmonella* strains, *Escherichia coli*, and *Clostridioides difficile*; use of phage endolysin or tail proteins; the effect of mucous or dopamine on phages; a salutary case report of phage therapy failure; use of machine learning and simulations to test phage therapy; and a review of regulatory hurdles for the use of phage.

Phages are versatile agents that can be used for various purposes, from food safety to human health. However, lack of comprehensive guidelines and regulations in many countries hinders their widespread applications. Karn et al. reviewed the use of phages in a variety of situations such as veterinary science, agriculture, food preservation and of course human health (for example bacteremia, gastrointestinal tract infections and respiratory tract infections) due to their high specificity, low toxicity and ability to adapt to bacterial mutations. However, their use has challenges such as their pharmacokinetics and pharmacodynamics and delivery. This paper also includes information on FDA-approved bacteriophage-based product, commercial phage product, and global list of companies using phages for therapeutic purposes. Whilst Karn et al. touched upon the regulatory challenges of phage therapy, this was discussed more in the review of Yang et al.. In Russia and Georgia, phage therapies can be purchased without prescription partly because they have been used frequently and for a long time in these countries. Phage therapy was classified as a medicinal product by European Medicines Agency, but different European countries regulate its use in different ways, with Belgium having “an established, innovative, and distinctive regulatory framework.” China, as many other countries, is developing regulatory systems as the importance of phage therapy is being recognized as a rapidly growing and important technology.

Two papers reviewed the potential and challenges of phage therapy for chronic airways disease, a condition characterized by persistent bacterial infections and neutrophilic inflammation. Phage therapy may help phagocytes of our innate immune system control chronic airways disease, although the Laucirica et al. concluded that the exact mechanisms underlining this require further investigation. As well, phages can adhere to the mucous, helping them remain in lungs, although whether this helps or hinders productive infection was considered by Ling et al. to be relatively understudied.

Phage therapy is a promising, but two original research papers highlighted the challenges it encountered. The study by Zhang et al. demonstrated that dopamine could change the structure of phages preventing them from infecting their host cells. This may have important implications especially as dopamine is being used to bind various moieties including antimicrobials to medical devices (6). Whilst these antimicrobial surfaces are designed to reduce medical device-associated infections, should infections occur, albeit more rarely, phage therapy for infections of such devices could fail. This deserves further research. The case report of Blasco et al. provided a reminder that phage therapy may not always progress as hoped. A patient with a recurring prosthetic vascular graft infection caused by *Pseudomonas aeruginosa* was treated with a phage cocktail initially alone and subsequently in combination with ceftazidime-avibactam. Another blood stream infection occurred after the phage therapy, although interestingly the *P. aeruginosa* isolated at that time had reverted to being susceptible to ß-lactams and quinolones, highlighting the beneficial trade-off effects of phage therapy, which could increase the susceptibility of bacteria to antibiotics (7).

Plunder et al. describes how *in silico* simulations may help tailor future therapeutic choices. The paper developed and tested a novel method that uses machine learning and multi-criterial optimization to find the optimal viral dose and administration time for phage therapy against bacterial infections.

Three Research Topic demonstrated the characteristics and applications of different phages and phage products for bacterial infections. Ahamed et al. characterized two lytic phages that targeted *Shigella flexneri, Shigella dysenteriae*, and *Shigella sonnei*. The phages individually or as a cocktail could reduce the numbers *Shigella* on raw chicken, indicating their potential use in the food industry, and as therapeutics for *Shigella* infection. However, there are many regulations, dose and stability issues that need to be addressed before phage therapy can be widely applied in the food industry. Jo et al. characterized a new jumbo phage that was active against antimicrobial resistant strains of *E. coli*. The phage, EJP2, was active against resistant and pathogenic strains of *E. coli*, as well as biofilms and had synergistic activity with cefotaxime (Jo et al.). Not all bacterial infections are immediately amenable to phage therapy. For example, Umansky and Fortier describe phages of *C. difficile* as usually being not lytic and so need to be engineered. Studies on the mechanism of action of phage products demonstrated that the tailspike proteins of epsilon 34 phage disrupted the membrane of the *Salmonella* Typhimurium and *Salmonella* Newington as well as causing reductions in the bacteria's dehydrogenase activity (Ibrahim et al.). Stevens et al. demonstrated that phage endolysin helped to treat a chronic *Enterococcus faecalis* prostate infection that was causing severe chronic pelvic pain syndrome associated with bacterial prostatitis.

While phages exhibit promising potential as alternatives for treating drug-resistant bacterial infections and have shown success in various pathologies, the limited fulfillment of clinical requests underscores challenges in comprehending phage biology, host interactions, and the necessity for standardized regulations to fully exploit the benefits of phage therapy.

## Author contributions

DS: Writing—review & editing. TF: Writing—review & editing. RB: Writing—review & editing. MW: Writing—original draft, Writing—review & editing.
